# Liver fibrosis-derived exosomal miR-106a-5p facilitates the malignancy by targeting SAMD12 and CADM2 in hepatocellular carcinoma

**DOI:** 10.1371/journal.pone.0286017

**Published:** 2023-05-25

**Authors:** Juan Hu, Cong Xie, Shangcheng Xu, Qinli Pu, Han Liu, Liping Yang, Wei Wang, Longchun Mao, Zhiqiang Li, Weixian Chen

**Affiliations:** 1 Department of Laboratory Medicine, The Second Affiliated Hospital of Chongqing Medical University, Chongqing, China; 2 The First Affiliated Hospital of Chongqing Medical and Pharmaceutical College, Chongqing, China; 3 Department of Neurology, Jiulongpo District People’s Hospital, Chongqing, China; The Hormel Institute (University of Minnesota), UNITED STATES

## Abstract

The mechanism of hepatocellular carcinoma (HCC) development induced by liver fibrosis is obscure. The objective of this study is to establish miRNAs from exosomes associated with liver fibrosis, and to identify potential biomarkers for the prediction of personalized clinical management effectiveness in HCC. Our research focused on miRNAs from exosomes and mRNA from liver fibrosis, which we found in the gene expression omnibus (GEO) database. Weighted gene co-expression network analysis (WGCNA) evaluated miRNAs from exosomes associated with liver fibrosis, and Wilcoxon analysis assessed differentially expressed mRNAs (DEGs) across liver fibrosis/normal tissues. Following that, DEGs were assessed through gene set enrichment analysis (GSEA), gene ontology (GO), and the Kyoto Encyclopedia of Genes and Genomes (KEGG). In addition, based on the screened targeted genes, including SAMD12 and CADM2, we further elucidated their correlation in HCC patients from the BEST database. The Kaplan-Meier Plotter platform was applied to evaluate the prognostic values of miRNA in HCC. In vitro and vivo experiments validated our findings. Six miRNAs associated with liver fibrosis were evaluated in our investigation. In-depth research presented exosome-derived miR-106a-5p, SAMD12 and CADM2 could exert valuable predictive implications for HCC treatment and illness assessment. Serum miR-106a-5p derived from liver fibrosis was decreased compared with healthy individuals. SAMD12 and CADM2 were diminished in liver cancer cell lines, and their knockdown of them exacerbated the proliferation capacities of liver cells in vitro. Exosome-derived miRNA of liver fibrosis modulated tumorigenesis by targeting SAMD12 and CADM2 in HCC.

## Introduction

The latest global cancer statistics showed that liver cancer was the third most common malignancy causing cancer mortality [1]. Patients with liver fibrosis were at high risk of developing primary liver cancer, specifically HCC [2]. Fibrosis of the liver reflected a pathology-based condition defined through excessive proliferation/aberrant release of extracellular matrix (ECM) components. It is a slow, dynamic process, with a complex sequence of cellular interactions, cytokines, extracellular matrix, and degradation enzymes [3]. Long-term chronic liver stimulation by a variety of damage factors could trigger the liver stellate cells (HSC) in hepatic sinusoids, which lead to a disequilibrium in the synthesis and degradation of collagen and other ECM components. This lead to structural abnormalities and dysfunction of the liver [4], which could promote the development of liver cancer. But to date, the mechanism of liver cancer development is not fully understood.

Exosomes were first identified in 1983 by Johnstone et al. in sheep reticulocytes [5]. Under an electron microscope, the exosomes are 50–150 nm in diameter and have a “discoid” or “cup-shaped” morphology [6]. Exosomes have biological activity; they consist of lipids, proteins, messenger RNAs, and microRNAs (miRNAs); and they function as information carriers, mediating intercellular communication and regulating target cell function [7]. Each cell is capable of generating this kind of extracellular vesicle [8]. Exosomes function in a variety of ways depending on the cell type of origin and are involved in a variety of processes such as immune reaction, intercellular communication, antigen presentation, cell migration, cell differentiation and metastasis [9]. They also contribute to the supplementary diagnosis of cardiovascular, central nervous system diseases and cancer, and have been linked to hepatic, renal and pulmonary disorders [10]. Exosomes can be released extracellularly and are a rich source for discovering potential blood biomarkers [11]. Serum exosomes containing miR-660-5p, miR-125a-5p, and miR-122 are apparently related to the advancement of liver fibrosis. Additionally, the downregulation of miR-122 stimulates the proliferation of hepatic stellate cells and increases the expression of a wide range of fibrosis markers [12].

The microRNAs, which were non-coding single-stranded (22 nucleotide-long), were produced by endogenous genes and regulated the level of genomic expression after transcription via mRNA-binding in plants/animals [13]. Research suggested that an individual miRNA could target multiple transcripts, with individual transcripts being targeted by a myriad of miRNAs [14]. Fibrosis is a momentous risk factor of HCC [15, 16], but how fibrosis exosome-derived miRNAs interrupted disease evolution and induced tumor formation were under exploitation.

In the study, we systematically discerned and analyzed disparate expression miRNAs sourced from liver fibrosis exosomes. Furthermore, we estimated clinical relevance and individualized therapeutic interventions of the targeted genes based on multi-omics data. We reasonably hypothesized that these miRNAs may be involved in the development of hepatic fibrosis by controlling the transcriptomic expression and further intervening in tumorigenesis. In vitro and vivo experiments demonstrated that targeted genes could disturb the tumorigenesis progression of HCC, and exosome-derived miR-106a-5p might exert a valuable role in diagnosing liver fibrosis. Our study unveiled the novel perception of SAMD12 and CADM2 and provided effective strategies for prospective management in HCC individuals.

## Materials and methods

### Data download

First, the GEO database was investigated for data sets related to liver fibrosis exosomes through keywords such as “liver fibrosis” and “exosomes”. Next, we downloaded GSE179961 and GSE171294. Regarding miRNA expression profiles linked with liver fibrosis-derived serum exosomes, 18 patients in various stages (0–4) of liver fibrosis were enrolled in GSE179961 (platform: GPL16791 Illumina HiSeq 2500). For gene/mRNA expression profiling related to liver fibrosis tissues in liver fibrosis, 4 normal liver tissue and 4 liver fibrosis patients were enrolled in GSE171294 (platform: GPL24676 Illumina NovaSeq 6000).

### miRNAs and DEG screening

For serum exosomes miRNA, some miRNAs (*P*-value < 0.05 and cor > 0.47) related to the progress of liver fibrosis were screened using “WGCNA”. Herein, the selected soft-threshold power was 10, while the correlation coefficient threshold was 0.8. For DEGs, the expression matrix (FPKM) from GSE171294 was analyzed using Wilcoxon. A *P*-value of 0.05 was deemed to be the statistical significance cut-off.

### Functional enrichment assessments

GSEA probed putative biological pathways in liver fibrosis using the clusterProfiler R tools (*P* < 0.05). Furthermore, the KEGG pathway and GO studies of DEGs both implemented cluster profiler R packages to forecast the likely roles of these DEGs. The GO assessment was carried out through multiple perspectives: biological process (BP), cellular component (CC), and molecular function (MF), with *P* < 0.05 values being statistically significant.

### Analysis of the correlation between miRNA and liver cancer

Based on the screened targeted genes, including SAMD12 and CADM2, we further elucidated their correlation in HCC patients from the BEST database (https://rookieutopia.com/app_direct/BEST/). In the prediction process, the parameters are subject to default. In addition, the Kaplan-Meier Plotter platform was applied to evaluate the prognostic values of miRNA in HCC.

### Statistical analysis

The log-rank test examined the KM analysis and Wilcoxon signed-rank test was utilized to determine the differences between diverse groups. The receiver operating characteristic (ROC) curves were carried out to identify the accuracy of parameters. *P* < 0.05 was considered as statistically significant difference.

### Sample collection

From March 2023 until April 2023, the project enrolled five healthy and liver fibrosis individuals. The liver fibrosis group consisted of individuals who exhibited abnormal ultrasound or radiological imaging findings or medical history, while the normal group did not present relevant anomalous behaviors. Patients who were treated previously were excluded from our study. This study was approved by the Institutional Ethics Committee for human studies at the Second Affiliated Hospital of Chongqing Medical University. All procedures followed the Declaration of Helsinki.

### Cell culture and transfection

The human normal liver cell line QSG-7701 and the human hepatic stellate cell line LX-2 were preserved in our laboratory. All cells were maintained in 5% CO_2_ at 37°C in Dulbecco’s modified Eagle medium (DMEM, Gibco, USA) supplement ed with 10% fetal bovine serum (Pan Seratech, Germany), 100 U/mL penicillin, and 100 μg/mL streptomycin. Transient transfections were performed with Lipofectamine 2000 (Invitrogen, Carlsbad, USA), according to the manufacturer’s protocol.

### Total RNA extraction and qRT-PCR analysis

Cells treated were lysed with Trizol (Invitrogen, Carlsbad, USA). Complementary single-stranded DNA (cDNA) was synthesized from total RNA by reverse transcription (TaKaRa, Japan). Quantification of cDNA targets was performed on CFX96TM Real-Time-PCR Detection System (Bio-Rad, USA), and the primers were synthesized by Sangon Biotech (Shanghai, China). The sequences are listed in S1 Table. Every sample was repeated 3 times using SYBR Green master mix (TaKaRa, Japan). The relative expression levels of the targeted gene were normalized to β-actin and calculated using the 2-ΔΔCt method.

### CCK-8

CCK-8 test was performed to measure the cell proliferation capacity. In brief, 2000 cells treated with transfection were cultured into 96-well plates for the indicated time. And 10 μL of CCK-8 (Dojindo Laboratories, Kumamoto, Japan) was added to each plate and incubated for 1 h. The absorbance was examined at 450 nm in triplicate.

### Western blot

The procedure was followed by prior study [17]. The protein signals were visualized using ECL (Millipore, Massachusetts, USA), and the signal strength was analyzed using Image Lab V6.0 software. The internal reference used was GAPDH. The primary antibodies were as follows: α-SMA (cat. no. ab5694, 1:1000), TGF-β (cat. no. ab179695, 1:1000), goat anti-rabbit second antibody (HRP cross-linking) (cat. no. ab205718, 1:1000) and GAPDH (cat. no. ab181602, 1:1000). All antibodies were purchased from Abcam (Cambridge, UK).

## Results

### miRNA/DEG screens

**Fig 1:**
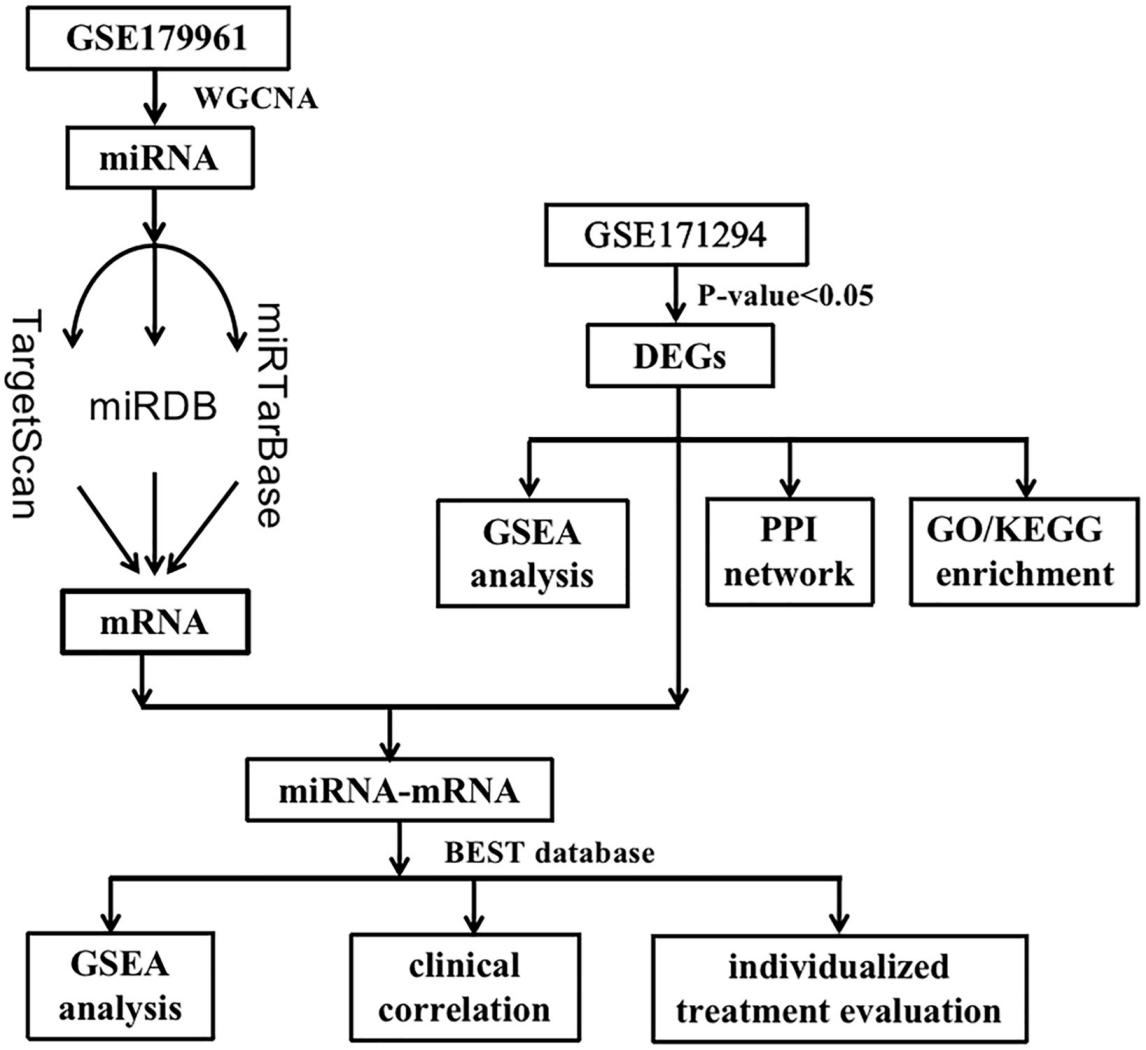
The workflow of this study.

**Fig 2:**
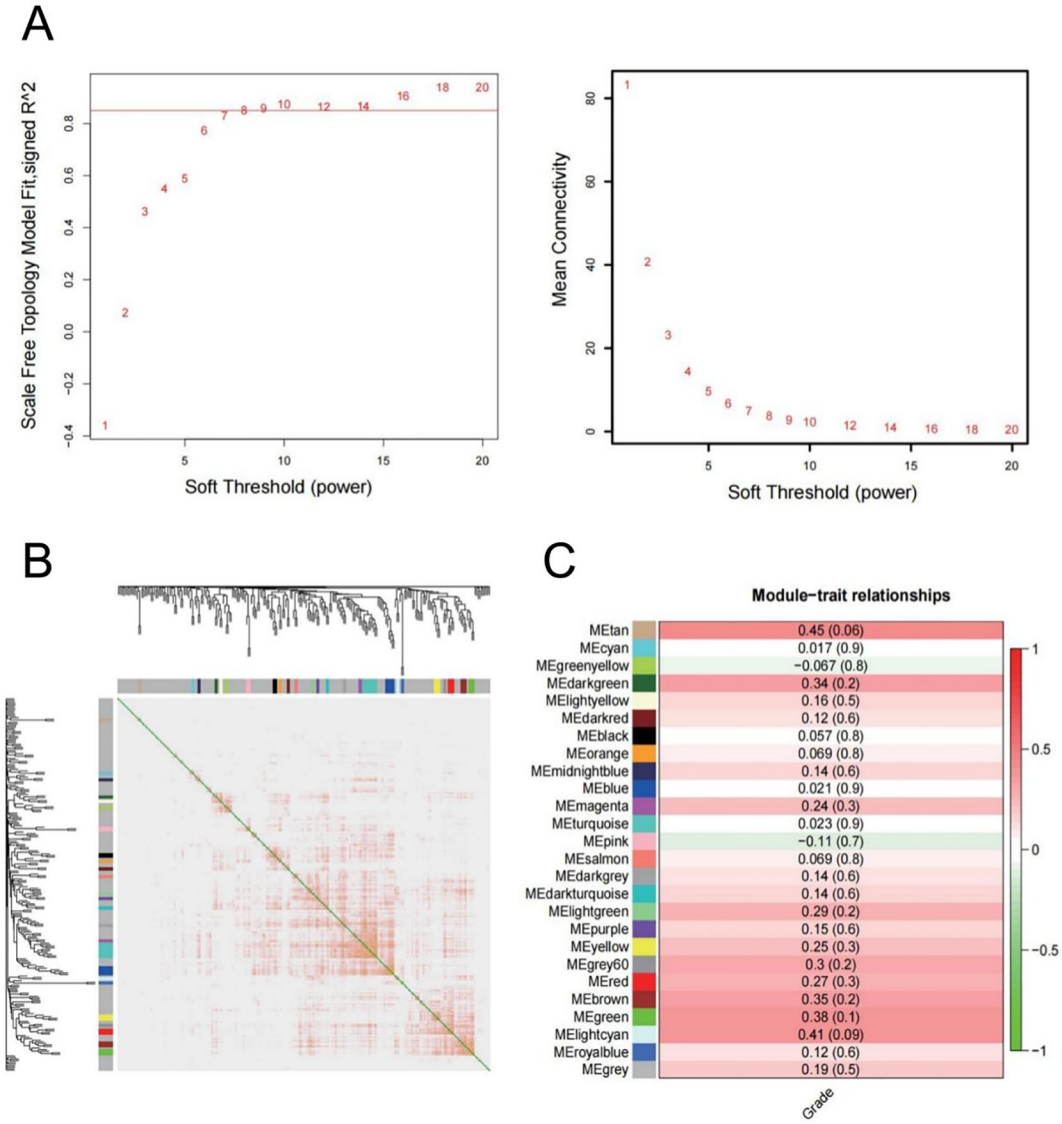
WGCNA analysis. (A) Analysis of the scale-free fit index for various soft-thresholding powers (Left) and analysis of the mean connectivity for various soft-thresholding powers (Right). (B) Network heatmap plot in the co-expression modules (The progressively saturated red colors indicated higher overlap among the functional modules.). (C) Module-trait relationships.

**Fig 3:**
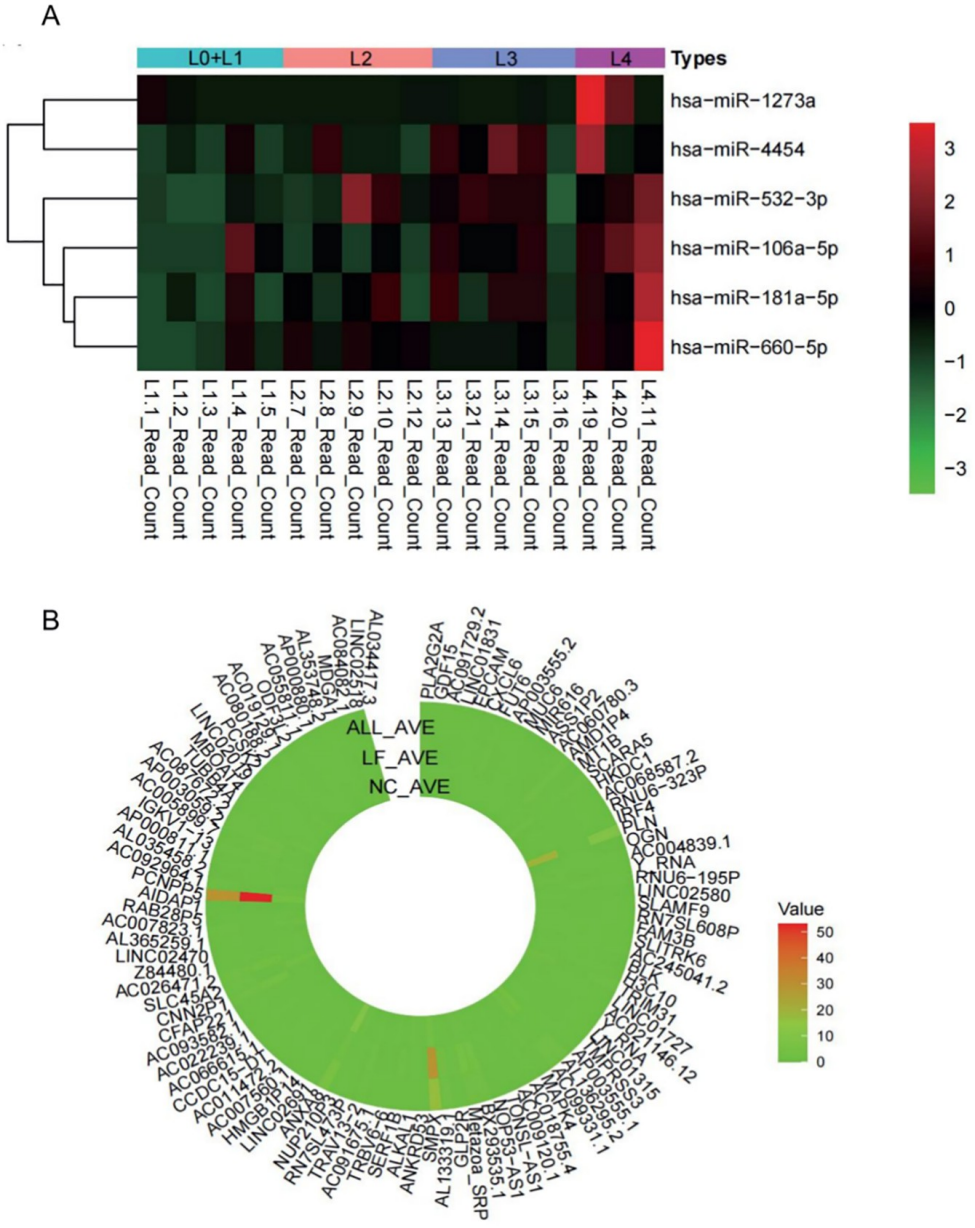
Heatmap plot. (A) miRNA expression level in GSE179961. (B) Top 100 DEGs expression level in GSE171294.

Data were assessed independently (as shown in Fig 1). For miRNAs (GSE179961), 6 miRNAs related to liver fibrosis were screened after WGCNA analysis (hsa-miR-106a-5p, hsa-miR-1273a, hsa-miR-181a-5p, hsa-miR-4454, hsa-miR-532-3p, and hsa-miR-660-5p, as illustrated in Figs 2 and 3A and Table 1). Regarding DEGs, 448 differentially expressed mRNAs (228 upregulated and 220 downregulated) within GSE171294. The 100 genes with the most obvious FC (upregulated or downregulated) were shown in Fig 3B. These miRNAs and DEGs were used for the next analysis.

**Table 1:** Six miRNAs related to liver fibrosis were screened from WGCNA analysis.

Grade	P-value	Cor
hsa-miR-106a-5p	0.014624155	0.564683916
hsa-miR-1273a	0.046841498	0.474104508
hsa-miR-181a-5p	0.016998561	0.5542267
hsa-miR-4454	0.03317443	0.50345342
hsa-miR-532-3p	0.033381213	0.502945373
hsa-miR-660-5p	0.023742005	0.529782238

### Functional enrichment analysis

**Fig 4:**
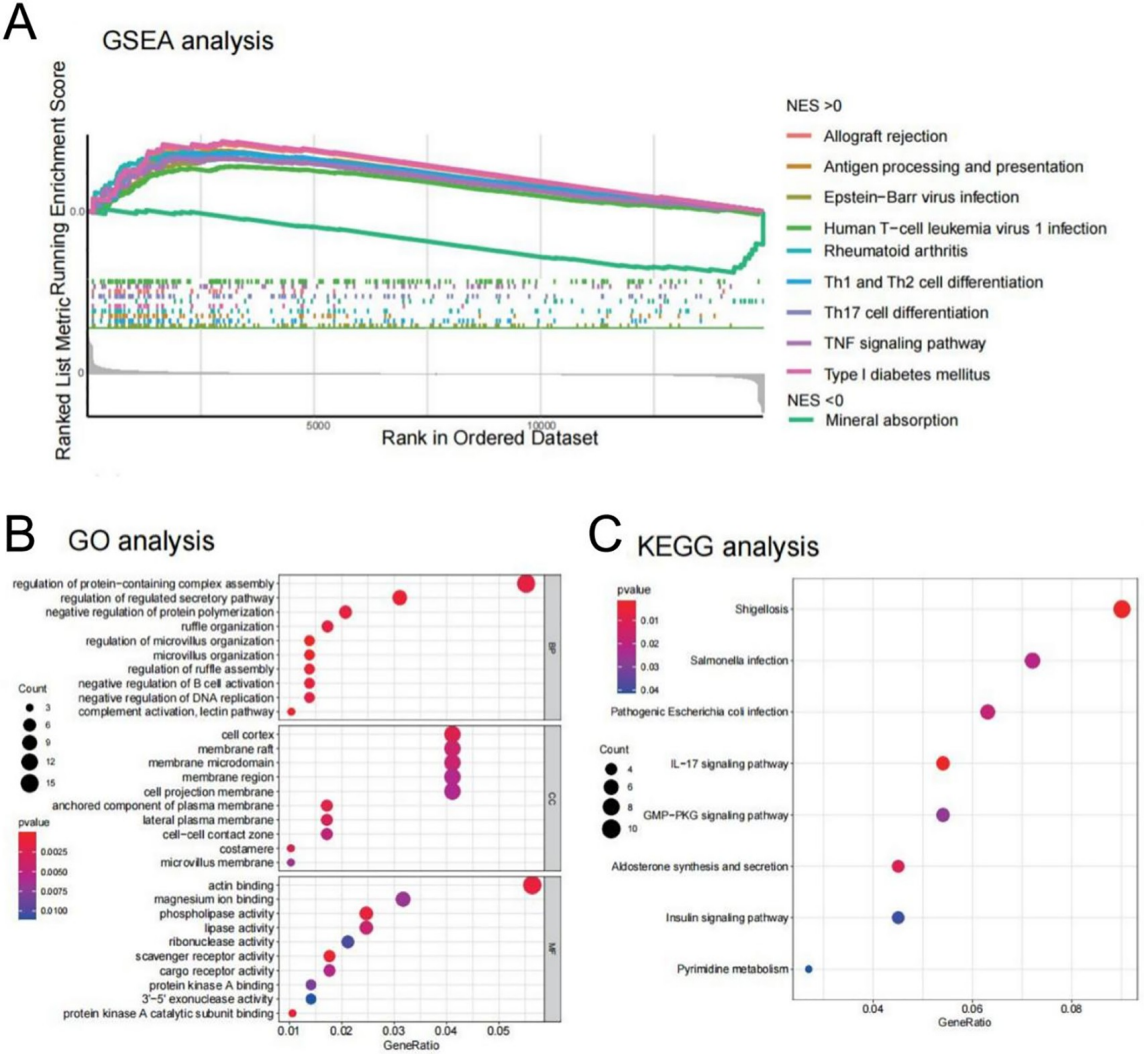
Function enrichment analysis of DEGs. (A) GSEA analysis. (B) GO analysis of DEGs. (C) KEGG analysis of DEGs.

Fig 4A depicted GSEA dataset analytical outcomes highlighting genes associated with immunity and inflammation (e.g., Allograft rejection, Antigen processing and presentation, Epstein-Barr virus infection).

BP, CC, together with MF were designated GO functional annotation categories. Fig 4B showed the top 30 enriched GO elements (The details of enriched GO elements were shown in S1 Table). The regulation of protein-containing complex assembly, regulation of the secretory pathway, and negative regulation of protein polymerization were all considerably enriched in these 448 DEGs, according to GO BP analysis. These 448 DEGs were found to be highly abundant in the cell cortex, membrane raft, membrane microdomain, membrane region, and cell projection membrane when CC analysis was performed. Actin binding, magnesium ion binding, and phospholipase activity were all examined by MF for these genes. The 448 DEGs were then subjected to a KEGG pathway enrichment analysis. The 448 DEGs were considerably enriched in the Shigellosis, Salmonella infection, and Pathogenic Escherichia coli infection, as shown in Fig 4C (The details of KEGG were shown in S2 Table).

### Analysis of the correlation between miRNA and liver cancer

**Fig 5:**
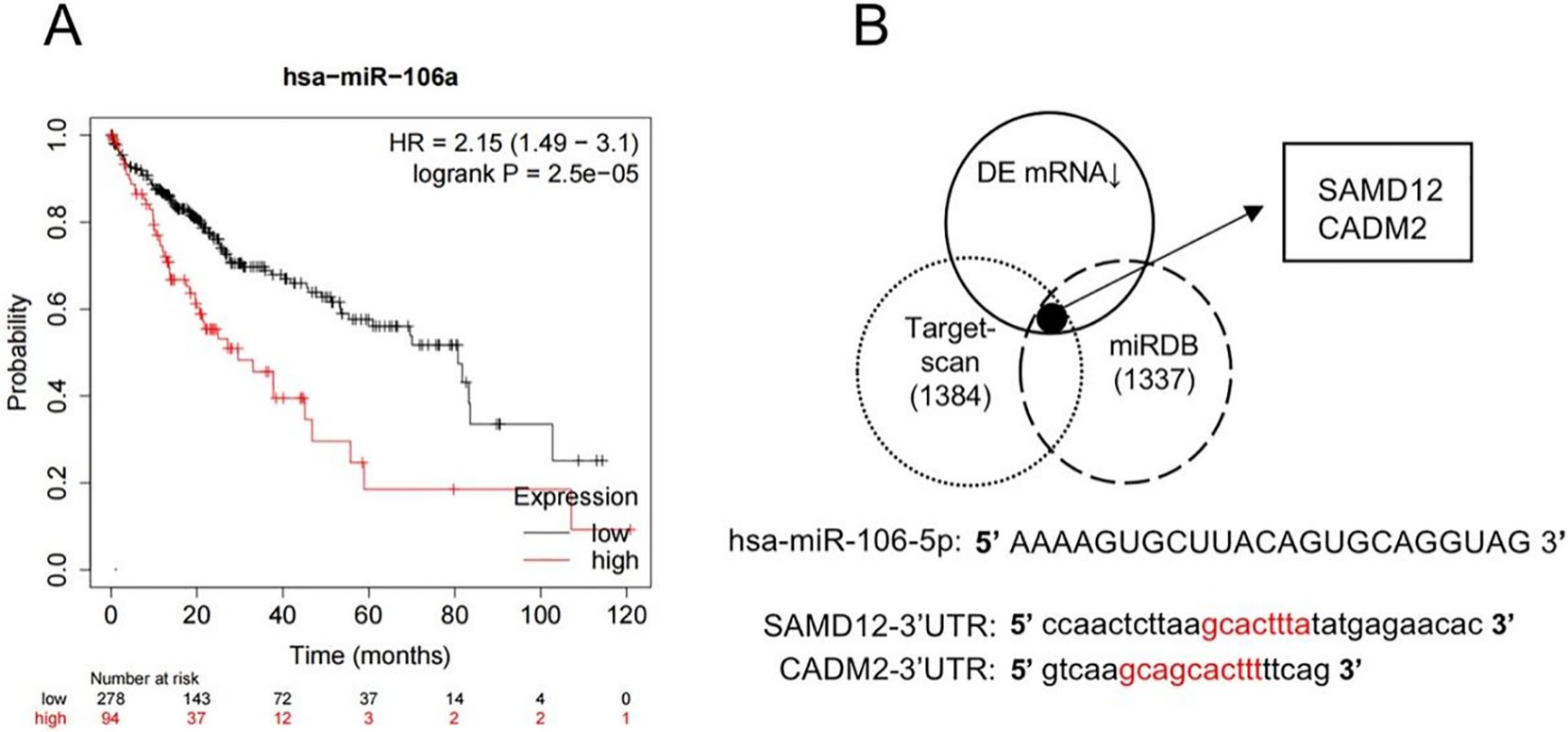
Prediction of targeted genes. (A) Survival analysis of miR-106a-5p in HCC patients. (B) Potential targeted genes of miR-106a-5p.

Given the enrichment results were predominantly related to tumor progression, we then analyzed the clinic correlation between the above miRNAs and HCC. As expected, miR-106a-5p was verified to be associated with poor outcomes in HCC (Fig 5A). Based on the DEGs and targeted genes predicted from two public databases, we determined two molecules, including SAMD12 and CADM2 (Fig 5B). As shown in Fig 5C, we speculated their potential combining sequences with miR-106a-5p.

### Molecular function and clinical properties of targeted genes in HCC

**Fig 6:**
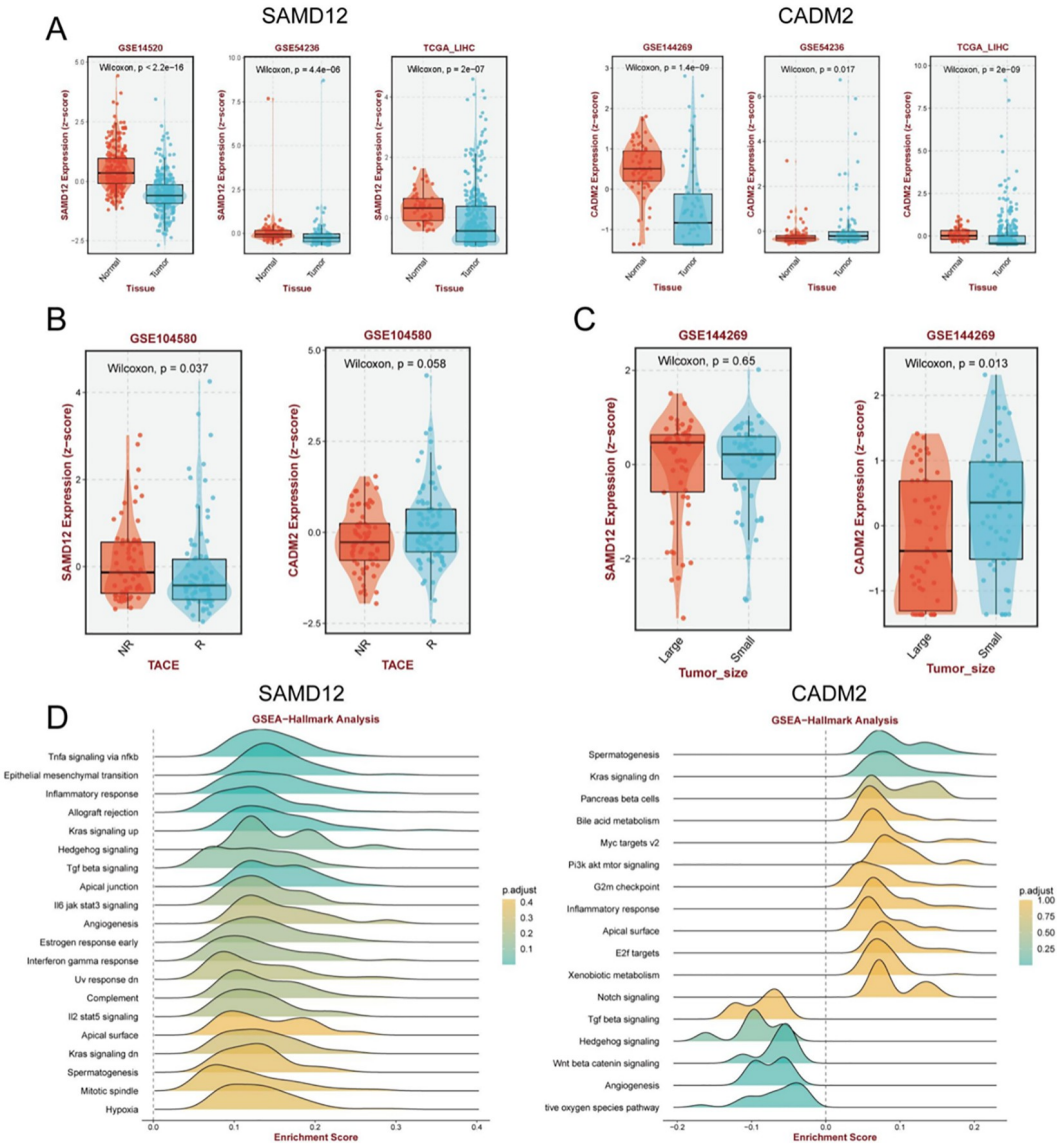
Clinical features of objective genes in HCC. (A) The expression difference in normal and tumor tissues. (B) Distinct levels in post-TACE surgery. (C) Diverse expression in different tumor sizes. (D) GSEA analysis of hallmark terms.

Subsequently, we investigated whether miR-106a-5p could facilitate the process of liver fibrosis to HCC by modulating the expression of SAMD12 and CADM2. We studied the clinical value of these two regulatory agents and their underlying pathogenesis. A number of external cohorts demonstrated that SAMD12 and CADM2 were down-regulated in HCC (Fig 6A). Transcatheter arterial chemoembolization (TACE) was implemented for intermediate-stage HCC patients. We also found a reduction of SAMD12 in the response group (*P* < 0.05, Fig 6B). Higher levels of CADM2 were found in small-volume tumors (*P* < 0.05, Fig 6C). Gene set enrichment analysis (GSEA) illustrated that SAMD12 and CADM2 were prominently enriched in diverse carcinogenic signaling (Fig 6D). These findings demonstrated that exosome-derived miR-106a-5p could potentially intervene liver fibrosis and HCC via SAMD12 and CADM2.

### Predictive importance of targeted genes in immunotherapy and chemotherapeutics

**Fig 7:**
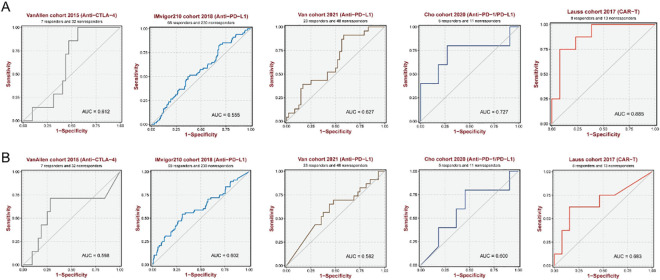
Predictive performance in clinical immunotherapy cohorts of (A) SAMD12 and (B) CADM2.

**Fig 8:**
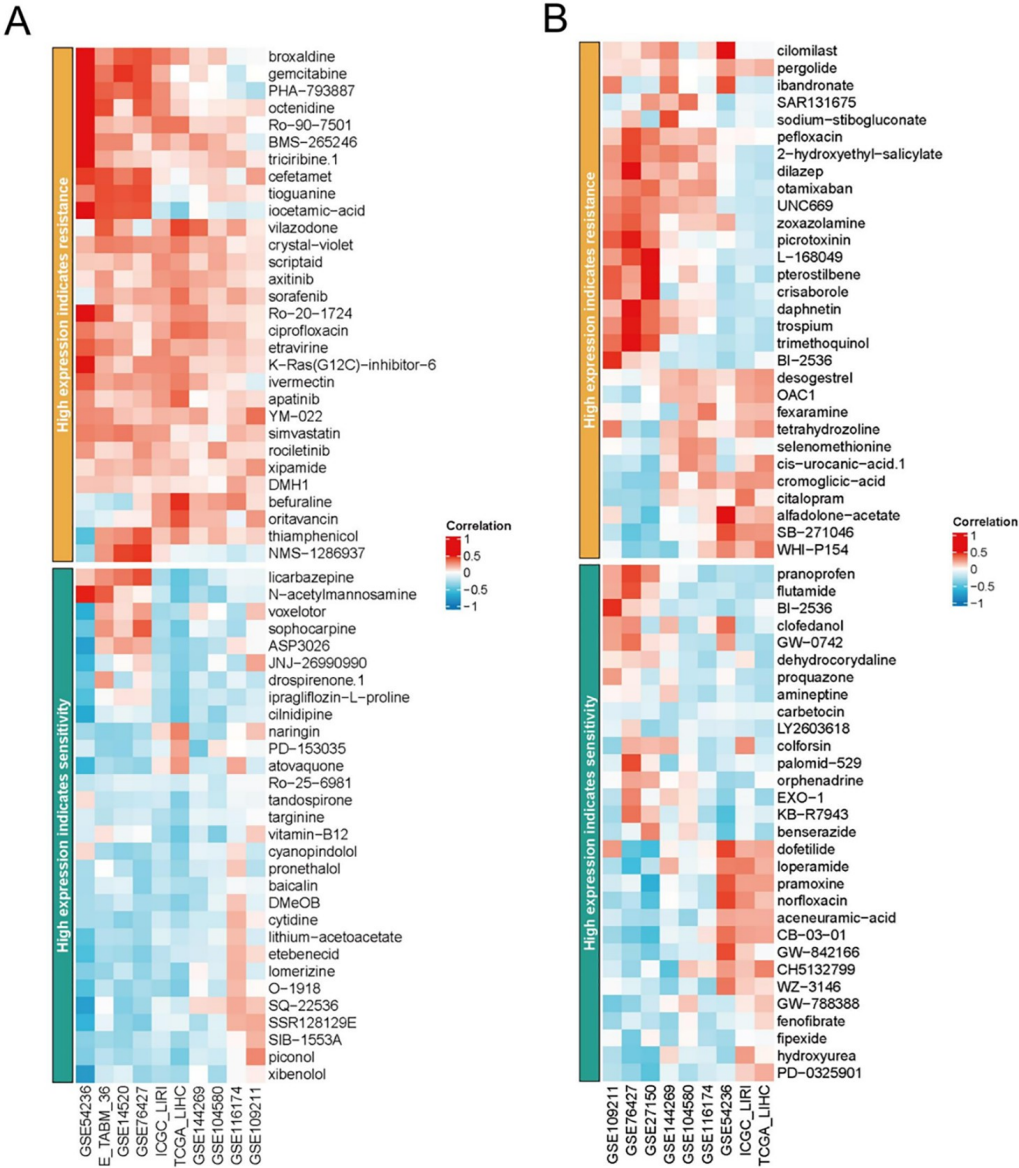
Guidance for regular chemotherapy agents sensibility of (A) SAMD12 and (B) CADM2.

Immune checkpoint inhibitors (ICIs) have transformed the landscape of tumor management, which could lead to a long-term response that would be beneficial to millions of patients. Nevertheless, we have not yet developed an index for monitoring therapeutic efficacy. Consequently, we examined five clinical immunotherapy cohorts and concluded that SAMD12 and CADM2 may be useful in predicting treatment for ICIs (Fig 7A and 7B). Even though most cancer treatments could reduce the growth of cancer, resistance to chemotherapy remained an important barrier to effectively treating HCC. In our research, we evaluated potential medications according to SAMD12 and CADM2 levels. Fig 8A showed that high SAMD12 indicated different therapeutic susceptibility, and Fig 8B illustrated that CADM2 could also be used as a guidance tool for clinical accuracy management. Our results suggested that exosome-originated miR-106a-5p could target SAMD12 and CADM2 to impact the performance of immunotherapy and chemotherapy, which could be applied as effective biomarkers for clinical diagnosis and prognosis.

### Biological validation in vitro and in vivo

**Fig 9:**
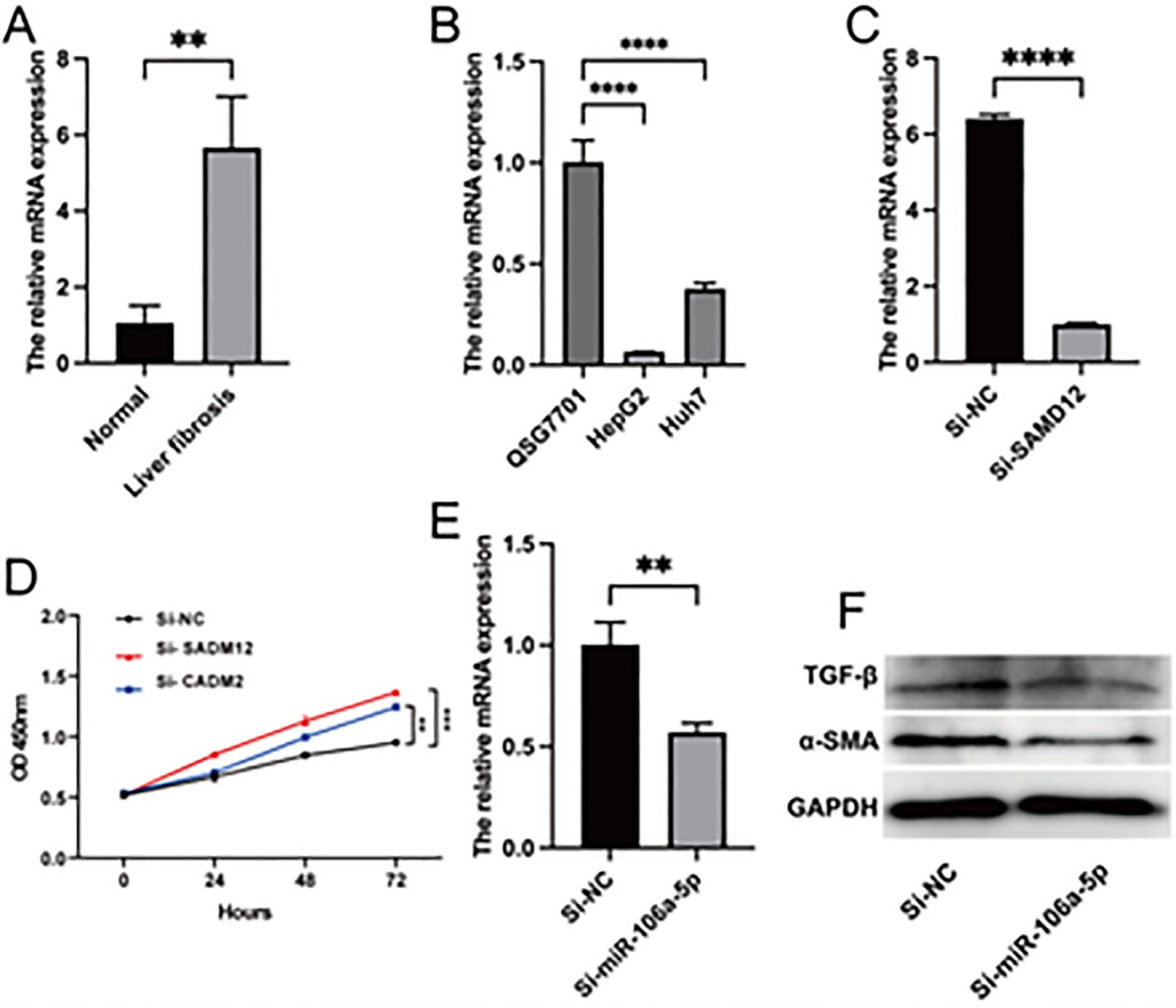
Biological validation in vitro and in vivo. (A) The levels of miR-106a-5p in patients with liver fibrosis. (B) SAMD12 mRNA levels in normal liver and liver cancer cells. (C) PCR validation of SAMD12 knockdown efficiency. (D) SAMD12 and CADM2 promote the ability of proliferation in QSG7701 cells. (E) PCR validation of miR-106a-5p knockdown efficiency. (F) Western blot revealed that miR-106a-5p could regulate the process of hepatic fibrosis in LX2 cells. Data represents the mean ± SD (N = 3).

To verify our findings, we evaluated the level of miR-106a-5p in healthy subjects and patients with liver fibrosis. As depicted in Fig 9A, miR-106a-5p was augmented in the liver fibrosis group. Knockdown of miR-106a-5p led to a decrease in the expression levels of α-SMA and TGF-β, indicating the involvement of miR-106a-5p in promoting the activation of fibroblasts (Fig 9E and 9F). Furthermore, to assess the molecular function of SAMD12 and CADM2, we silenced them in liver cells and implemented a CCK-8 assay to detect their impact on cell proliferation. Our results uncovered that down-regulated SAMD12 and CADM2 exacerbated the proliferation potency, demonstrating the tumor suppression potential of SAMD12 and CADM2 in liver tumors (Fig 9B–9D). And our experiments demonstrate that CADM2 may be less effective due to low basal expression (S1 Fig).

## Discussion

The development of liver cancer is an extremely complex and dynamic process, it may be associated with various cellular signaling pathways, exosomes, Toll-like receptors, autophagy, and several other factors [18]. Intercellular crosstalk among distinct liver cells played an imperative role in liver fibrosis, which could shape the organizational microenvironment and modulate the immune response to the tumor originating within itself [19, 20]. Besides, liver fibrosis is a vital driver of tumor immune escape and encroaches the challenges facing the application of ICIs [21]. Most HCC patients progressed from a collection of chronic liver injury, hepatic inflammation, and fibrosis [22]. Notably, transcriptional and posttranscriptional regulation are critical for HCC pathogenesis. Therefore, it is significant to study the molecular mechanism of hepatic fibrosis in order to find a new therapeutic target and to facilitate remedies for HCC patients.

The regulatory network controlling serum exosomes produced from liver fibrosis was identified in our study using an integrated analysis of microarray and RNA-sequencing data sets. Four hundred and forty-eight DEmRNAs and six DEmiRNAs were identified as major factors in liver fibrosis. NRBP1, SAMD12, TAB3, CADM2, and CBX4 were identified as hub genes in the miRNA-mRNA network using the 448 DEmRNAs. The 5 RNAs were identified as biomarkers in serum exosomes of patients with liver fibrosis, a finding that needs to be further tested in clinical settings.

Based on the predicted targeted genes, we further analyzed their molecular functions. SAMD12 and CADM2 may play an alternate role in postoperative effects and tumor size, possibly contributing to carcinogenesis. ICIs, combined with routine chemotherapy, have improved the quality of care in a subset of patients with advanced disease [23, 24]. SAMD12-AS1 promotes gastric cancer progression via the DNMT1/p53 axis [25], and HBV-encoded HBx promotes HCC development through enhanced SAMD12-AS1 transcription [26]. In hepatocellular carcinoma cells, miR-10b directly targets CADM2. miR-10b/CADM2 regulates hepatocellular carcinoma cells’ EMT activity, together with migratory capabilities through the focal adhesion kinase (FAK)/AKT signaling pathway [27]. Additionally, it enhances cerebral metastases within non-small cell lung cancer cases by triggering epithelial-mesenchymal transition (EMT) [28] CADM2 suppresses glioma growth, migration, and invasion [29]. Nevertheless, the low likelihood of response and drug resistance remarkably affected the expected results [30, 31]. And there is a pressing need to develop novel parameters for forecasting therapeutic effectiveness and accurate management. In accordance with the validation of various clinical immunotherapy cohorts, we revealed that SAMD12 and CADM2 may be reliable predictors of immunotherapy and chemotherapy efficacy in our research, which may prove to be useful tools for individualized therapy.

However, there are certain shortcomings in our present research. First of all, although this bioinformatics activity has been well organized and has set a strict threshold for WGCNA analysis, it has not been validated in vivo or in vitro. Additionally, the study was retrospective research, as it used data from the GEO data set without conducting a prospective clinical trial to validate the findings. Despite these limitations, the current research gives a comprehensive view of the miRNAs driving exosomes originating from liver fibrosis, which might shed new light on the underlying molecular mechanisms of the initiation and formulation of HCC, and recognized novel biomarkers for its evaluation and accurate healthcare.

## Conclusion

We applied a comprehensive analysis of two RNA-seq data sets to identify microRNAs that could have pivotal parts in generating hepatic fibrosis-derived exosomes. Additionally, we developed exosome-derived miR-106a-5p and its targeted genes, SAMD12 and CADM2 could act as applicable indicators for HCC individualized care and the inspection of the disease condition.

## Supporting information

S1 Fig The relative mRNA expression. (A) CADM2 mRNA levels in normal liver cells and liver cancer cells. (B) PCR validation of CADM2 knockdown efficiency. Data represents the mean ± SD (N = 3).
(TIF)

S1 Table GO analysis of DEGs (TOP.10). (DOCX)

S2 Table KEGG analysis of DEGs (TOP.8). (DOCX)

S3 Table Primer and SiRNA sequences. (DOCX)
