# Supplementary figures and images for: Liver fibrosis-derived exosomal miR-106a-5p facilitates the malignancy by targeting SAMD12 and CADM2 in hepatocellular carcinoma
Source: PLoS One. 2023 May 25;18(5):e0286017. doi: 10.1371/journal.pone.0286017 (PMC10212176; doi:10.1371/journal.pone.0286017)

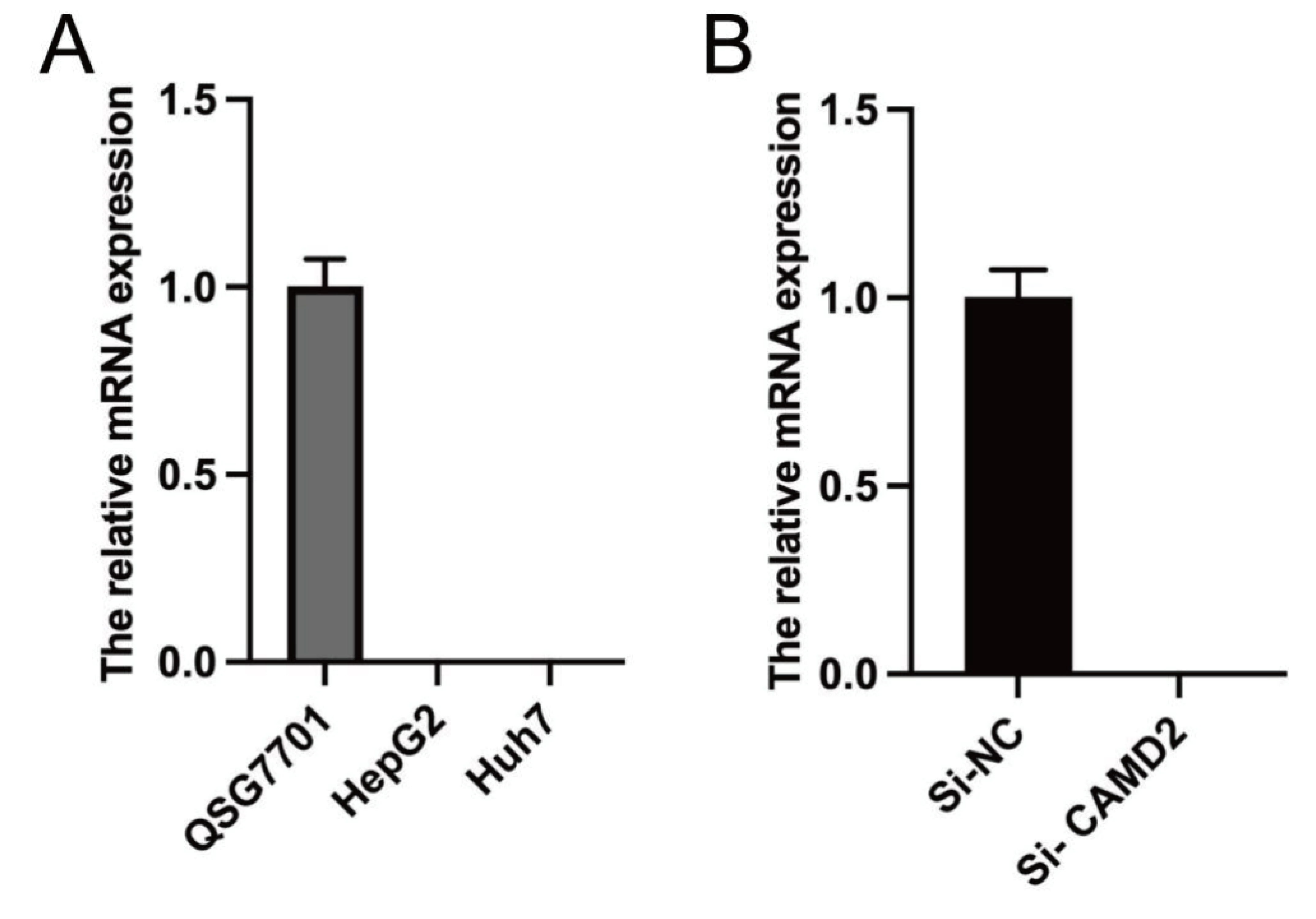

Supplement: S1 Fig — (A) CADM2 mRNA levels in normal liver cells and liver cancer cells. (B) PCR validation of CADM2 knockdown efficiency. Data represents the mean ± SD (N = 3). (TIF) [file pone.0286017.s001.tif]
