# Supplementary material for: Liver fibrosis-derived exosomal miR-106a-5p facilitates the malignancy by targeting SAMD12 and CADM2 in hepatocellular carcinoma
Source: PLoS One. 2023 May 25;18(5):e0286017. doi: 10.1371/journal.pone.0286017 (PMC10212176; doi:10.1371/journal.pone.0286017)
Supplement: S1 Table — (DOCX) [file pone.0286017.s002.docx]

**S1 Table: GO analysis of DEGs (TOP.10)**

| Ontology | Description | *P*value | Count |
| --- | --- | --- | --- |
| BP | regulation of microvillus organization | 0.000035050 | 4 |
|  | microvillus organization | 0.000536176 | 4 |
|  | complement activation, the lectin pathway | 0.000713533 | 3 |
|  | regulation of the regulated secretory pathway | 0.000921695 | 9 |
|  | regulation of ruffle assembly | 0.001093558 | 4 |
|  | negative regulation of protein polymerization | 0.001360848 | 6 |
|  | negative regulation of B cell activation | 0.001400776 | 4 |
|  | regulation of protein-containing complex assembly | 0.001664913 | 16 |
|  | ruffle organization | 0.001669199 | 5 |
|  | negative regulation of DNA replication | 0.001763559 | 4 |
| CC | anchored component of the plasma membrane | 0.002001817 | 5 |
|  | cell cortex | 0.002213146 | 12 |
|  | costamere | 0.002672981 | 3 |
|  | lateral plasma membrane | 0.002850232 | 5 |
|  | membrane raft | 0.004079805 | 12 |
|  | membrane microdomain | 0.004178886 | 12 |
|  | cell-cell contact zone | 0.004987972 | 5 |
|  | membrane region | 0.005651852 | 12 |
|  | cell projection membrane | 0.005780345 | 12 |
|  | microvillus membrane | 0.006640974 | 3 |
| MF | scavenger receptor activity | 0.000772367 | 5 |
|  | protein kinase A catalytic subunit binding | 0.000934841 | 3 |
|  | phospholipase activity | 0.001235225 | 7 |
|  | actin binding | 0.001347091 | 16 |
|  | lipase activity | 0.004141112 | 7 |
|  | cargo receptor activity | 0.005463194 | 5 |
|  | magnesium ion binding | 0.006833614 | 9 |
|  | protein kinase A binding | 0.007931601 | 4 |
|  | ribonuclease activity | 0.010141727 | 6 |
|  | 3'-5' exonuclease activity | 0.010979833 | 4 |
