# Supplementary material for: Liver fibrosis-derived exosomal miR-106a-5p facilitates the malignancy by targeting SAMD12 and CADM2 in hepatocellular carcinoma
Source: PLoS One. 2023 May 25;18(5):e0286017. doi: 10.1371/journal.pone.0286017 (PMC10212176; doi:10.1371/journal.pone.0286017)
Supplement: S2 Table — (DOCX) [file pone.0286017.s003.docx]

**S2 Table: KEGG analysis of DEGs (TOP.8)**

| Description | *P*-value | Count |
| --- | --- | --- |
| IL-17 signaling pathway | 0.001778848 | 6 |
| Shigellosis | 0.002015334 | 10 |
| Aldosterone synthesis and secretion | 0.010969748 | 5 |
| Pathogenic Escherichia coli infection | 0.018406094 | 7 |
| Salmonella infection | 0.020835484 | 8 |
| cGMP-PKG signaling pathway | 0.027013749 | 6 |
| Insulin signaling pathway | 0.039820212 | 5 |
| Pyrimidine metabolism | 0.041192551 | 3 |
