# Supplementary material for: Liver fibrosis-derived exosomal miR-106a-5p facilitates the malignancy by targeting SAMD12 and CADM2 in hepatocellular carcinoma
Source: PLoS One. 2023 May 25;18(5):e0286017. doi: 10.1371/journal.pone.0286017 (PMC10212176; doi:10.1371/journal.pone.0286017)
Supplement: S3 Table — (DOCX) [file pone.0286017.s004.docx]

**S3 Table: Primer and SiRNA sequences**

| Name | Sequences (5’→3’) |
| --- | --- |
| F: CADM2 | AAACTTCCAAGGCATATCTCACC |
| R: CADM2 | TGCGATTTGCATCCTCTTCTT |
| F: SAMD12 | TGCCCATGCTGAAGGTATTAAAC |
| R: SAMD12 | CGTAGCTGACTTAGCCGTCT |
| F:miR-106a-5p | GCACGTCCAAAAGTGCTTACAGT |
| R: miR-106a-5p | ATCCAGTGCAGGGTCCGAGG |
| RT-miR-106a-5p | GTCGTATCCAGTGCAGGGTCCGAGGTATTCGCACTGGATACGACCTACCT |
| F:U6 | AGAGAAGATTAGCATGGCCCCTG |
| F: U6 | ATCCAGTGCAGGGTCCGAGG |
| RT-U6 | GTCGTATCCAGTGCAGGGTCCGAGGTATTCGCACTGGATACGACAAAATA |
| Si-SAMD12 | GUGGAAUCUCAAUCCAUUAAATT |
| Si-CADM2 | GACAAUAGGAUCGAGCUGGUUTT |
| Si-NC | UUCUCCGAACGUGUCACGUTT |
